# Impression Cups

**Published:** 1883-09

**Authors:** 


### IMPRESSION CUPS.

Some years since Dr. Gunning devised peculiar forms of trays for obtaining impression of cases demanding the application of the inter-dental splint. They have never been kept in stock at the dental depots, and their existence was unknown to many. Lately, Dr. J. A. Bishop, of New York, believing that they would be found useful in many ordinary cases, had a number of them made by the

Meriden Britannia Metal Co., and they may now be obtained by writing to Dr. Bishop. They are deeper than the ordinary lower impression cup, and are perforated. They will be found especially convenient in the use of the modeling compounds, which so readily leave the cup when in the mouth.

